# Perception of Emotion in Conversational Speech by Younger and Older Listeners

**DOI:** 10.3389/fpsyg.2016.00781

**Published:** 2016-05-31

**Authors:** Juliane Schmidt, Esther Janse, Odette Scharenborg

**Affiliations:** ^1^Centre for Language Studies, Radboud UniversityNijmegen, Netherlands; ^2^International Max Planck Research School for Language SciencesNijmegen, Netherlands; ^3^Donders Institute for Brain, Cognition and BehaviourNijmegen, Netherlands

**Keywords:** affective speech, age, hearing sensitivity, natural speech, acoustic cues

## Abstract

This study investigated whether age and/or differences in hearing sensitivity influence the perception of the emotion dimensions arousal (calm vs. aroused) and valence (positive vs. negative attitude) in conversational speech. To that end, this study specifically focused on the relationship between participants’ ratings of short affective utterances and the utterances’ acoustic parameters (pitch, intensity, and articulation rate) known to be associated with the emotion dimensions arousal and valence. Stimuli consisted of short utterances taken from a corpus of conversational speech. In two rating tasks, younger and older adults either rated arousal or valence using a 5-point scale. Mean intensity was found to be the main cue participants used in the arousal task (i.e., higher mean intensity cueing higher levels of arousal) while mean *F*_0_ was the main cue in the valence task (i.e., higher mean *F*_0_ being interpreted as more negative). Even though there were no overall age group differences in arousal or valence ratings, compared to younger adults, older adults responded less strongly to mean intensity differences cueing arousal and responded more strongly to differences in mean *F*_0_ cueing valence. Individual hearing sensitivity among the older adults did not modify the use of mean intensity as an arousal cue. However, individual hearing sensitivity generally affected valence ratings and modified the use of mean *F*_0_. We conclude that age differences in the interpretation of mean *F*_0_ as a cue for valence are likely due to age-related hearing loss, whereas age differences in rating arousal do not seem to be driven by hearing sensitivity differences between age groups (as measured by pure-tone audiometry).

## Introduction

Accurate emotion recognition is a crucial component of successful social interaction (Blair, 2003). One modality in which affective information is conveyed is speech. Affect in speech manifests itself via differences in prosodic, acoustic patterns, which are used by listeners to derive the emotion intended by the speaker (Banse and Scherer, 1996; Scherer, 2003; Coutinho and Dibben, 2013). Studies have shown that the perception of affect is influenced by age (Orbelo et al., 2005; Paulmann et al., 2008; Mitchell et al., 2011). For instance, Paulmann et al. (2008) have shown that young adults are significantly better at recognizing the emotion categories anger, disgust, fear, happiness, and sadness from prosodic, acoustic information than middle-aged adults. Middle-aged adults in turn outperform older adults in recognizing emotion categories from affective prosody (Kiss and Ennis, 2001). The current study investigates where this age difference originates.

Important acoustic cues for affect perception include pitch, intensity, and articulation rate. Pitch is considered the most telling component of affective prosody (Mozziconacci, 1998; Hammerschmidt and Jürgens, 2007; Rodero, 2011). Emotions with higher levels of arousal, such as excitement, fear, and anger, have been shown to have higher mean *F*_0_ (Mozziconacci, 1998; Schröder, 2006). Other acoustic cues that signal to affective prosody are temporal aspects (Mozziconacci and Hermes, 2000), intensity (Schröder et al., 2001; Aubergé and Cathiard 2003), and spectral measures (Schröder, 2006; Tamarit et al., 2008). Importantly, these acoustic parameters are mutually dependent in speech, e.g., spectral measures such as spectral slope reflect the energy distribution over the spectrum and correlate highly with intensity (cf., Banse and Scherer, 1996). Moreover, intensity shows a strong positive correlation with pitch (cf., Hammerschmidt and Jürgens, 2007). As a consequence, acoustic patterns conveying affect in speech may be complex.

Aging affects the perception of these acoustic cues. Older adults, even those without hearing loss, have been reported to be less sensitive to pitch differences (He et al., 2007; Souza et al., 2011; Mitchell and Kingston, 2014), intensity differences (Harris et al., 2007), and temporal differences (Gordon-Salant and Fitzgibbons, 1999; Anderson et al., 2012) than younger adults. Given that these acoustic cues (pitch, intensity, and tempo) have been argued to convey affect in speech (Banse and Scherer, 1996), it might be hypothesized that the observed age effects in affect perception have their origins in the difference in the perception of acoustic cues compared to younger adults (but cf. Dupuis and Pichora-Fuller, 2015). However, few researchers have looked into the relationship between the use or interpretation of affect-related acoustic information and age-related differences in affective prosody perception. An exception is a study by Lima et al. (2014) who investigated affect perception in vocalizations without verbal content.

Additionally, many other causes have been proposed to explain the apparent age difference in verbal affect perception. Examples are general age differences in cognitive abilities, emotion regulation, and personality (Orbelo et al., 2005; Lambrecht et al., 2012; Lima et al., 2014). However, these cognitive or personality measures revealed either no effect (Lambrecht et al., 2012; Lima et al., 2014) or only a marginal effect (Orbelo et al., 2005) on differences in affect perception among participants. Prosodic emotion perception may be impaired at an auditory processing level (Mitchell and Kingston, 2014), including hearing loss. As the perception of acoustic cues such as intensity differences may be impacted by (high-frequency) hearing loss (cf., Boettcher et al., 2001), age-related hearing loss might moderate affect perception. Importantly, previous studies either have not included a large-enough range of hearing losses (Lambrecht et al., 2012; Schmidt et al., 2014) or have not related individual hearing loss to the acoustic properties of the stimuli (Orbelo et al., 2005; Dupuis and Pichora-Fuller, 2015).

In this study, we investigate the question whether age differences in the perception of affect-related acoustic cues can explain the observed age differences in the perception of affect in verbal stimuli. The first research question of this study is therefore whether younger and older listeners differ in their use or interpretation of acoustic cues for rating affect, and whether such a difference in cue use can explain the observed age difference in affect perception. This question is investigated by comparing the associations between acoustic parameters and affective ratings of a younger and an older listener group. We focus on three acoustic parameters: mean *F*_0_ (pitch cue), mean intensity (intensity cue), and articulation rate (tempo cue). These parameters are selected as they have been found to be important conveyers of affect and because sensitivity to pitch, intensity, and temporal changes has been found to be age-dependent. Moreover, in addition to the speech fragments’ absolute intensity, which may have been related to how far the speaker happened to be away from the microphone, we include a spectral measure related to vocal effort (Sluijter and Heuven, 1996), i.e., the Hammarberg Index (Hammarberg et al., 1980).

The second research question addressed in this study revisits the question whether hearing sensitivity plays a role in affect perception. As noted above, several earlier studies, where affect perception is mostly operationalized as emotion categorization performance, have suggested that age differences in affect perception should not be attributed to age-related decline in auditory abilities (e.g., Dupuis and Pichora-Fuller, 2015). Our study addresses this question by investigating the link between hearing sensitivity and differences in the perception of acoustic cues using a group of older listeners with a wide range of hearing sensitivity. We restrict this study to older adults who are not using hearing aids yet, even though some qualify for them.

In order to relate acoustic cues signaling affect to an individual’s perception of affect, researchers have frequently used two approaches: the categorical and the dimensional approach. In the categorical approach, concrete terms such as *happy*, *sad*, *neutral*, *bored*, or *angry* are used to describe different affectively colored utterances. Note, however, that the underlying affect concepts and interpretation of emotion terms may vary between individuals (Scherer, 2003, 2005). This is because category labels are numerous and require linguistic interpretation, i.e., higher level processing. The dimensional approach offers a more flexible and continuous description of affect (Wundt, 1905). Here, emotions are described by a two- or three-dimensional space, where the most frequently used axes are arousal (calm-aroused) and valence (negative-positive). Moreover, ratings do not depend on consistent interpretations of linguistic labels (such as *bored* or *angry*) because emotion dimensions are few, comprehensible, and easy to communicate to a participant in a linguistic (e.g., Likert scale, Likert, 1932) or non-linguistic manner (e.g., a pictorial self-assessment manikin, Bradley and Lang, 1994). In addition, the acoustic parameters pitch, intensity, and tempo have been shown to correlate with specific emotion dimensions (see, e.g., Sauter et al., 2010; Lima et al., 2013). Sauter et al. (2010) investigated the acoustics of non-verbal vocalizations and found, for instance, that arousal ratings were predicted by durational, pitch, and spectral measures. Therefore, the dimensional approach is employed in this study.

Unlike many other studies on age differences in affect perception, we investigate affect perception using natural stimuli rather than acted stimuli (e.g., Orbelo et al., 2005; Paulmann et al., 2008; Lambrecht et al., 2012). Ecological validity is highest in natural affective speech stimuli (Scherer, 2003) but the use of natural stimuli has certain difficulties and has consequently been little used to investigate affect perception. Stimuli taken from a natural speech corpus generally vary not just in affect dimensions, but also in semantic meaning, utterance length, and inter-speaker variations. Consequently, stimulus numbers might be relatively small after controlling for these factors. Additionally, natural speech corpora often have poor recording quality. A means to reduce this variability is to use controlled stimuli such as manipulated or acted speech, which has its own drawbacks. The encoding of verbal affect, particularly in acted speech, may be more extreme and prototypical (Scherer, 1986; Wilting et al., 2006) compared to natural speech in which affect perception is cued more subtly. Consequently, responses to natural and acted speech may differ, with more extreme affect realizations in the latter leading to more extreme responses (Wilting et al., 2006). There is evidence that listeners are indeed sensitive to the authenticity of affect. McGettigan et al. (2015) showed that listeners show different neural responses to authentic amusement laughter compared to more controlled voluntary laughter. Moreover, prototypical acoustic patterns with exaggerated frequency contours may be relatively easy to perceive for people with hearing loss (Grant, 1987). Consequently, hearing loss might be less predictive of changes in the use of acoustic cues if emotions are cued prototypically.

In short, this study investigates the perception of affective utterances in younger and older adults using a dimensional approach and natural (i.e., non-acted) speech stimuli by linking acoustic parameters and individual hearing loss directly to participants’ affective ratings. By doing so, we aim to investigate the origin of age differences in the perception of affect.

## Materials and Methods

### Participants

Two groups of participants were recruited to participate in the experiment: one younger group of students, and one older group. The younger group consisted of 20 native Germans who were students at Radboud University, Nijmegen (18 women, 2 men; age: *M* = 22.1 years, *SD* = 1.6, range: 19–24 years). The older group consisted of 20 native Germans who were recruited from the greater area of Saarbruecken via local senior clubs. All participants were paid for their participation. Older participants completed the Montreal Cognitive Assessment Test (MoCA), a brief cognitive screening test in order to check for mild cognitive impairment (Nasreddine et al., 2005). Two participants were excluded because they had a MoCA score of 20 or lower (out of 30; Waldron-Perrine and Axelrod, 2012). None of the participants used hearing aids in daily life. All participants underwent a hearing sensitivity test. Pure-tone thresholds for octave frequencies were measured for both ears with an Oscilla USB-300 PC-based screener audiometer (air conduction thresholds only). Individual pure-tone averages (PTA) over participants’ thresholds for 0.5, 1, 2, and 4 kHz in the better ear were used as an index of hearing sensitivity in the statistical analyses. Higher PTA indicated poorer hearing sensitivity. One older adult reported to have tinnitus and was therefore excluded from the analyses. The final group of older adults then consisted of 17 individuals (14 women, 3 men; age: *M* = 72.6 years, *SD* = 5.4, range: 61–82 years). There is evidence that gender differences exist in affect perception (e.g., Lambrecht et al., 2014). It is therefore important that both age groups have a similar distribution of female and male participants, with both age groups being skewed toward female participants. Mean PTA was 2.2 dB HL (*SD* = 3.8, range: -5.0 to 10.0 dB HL) for the younger listener group and 25.1 dB HL for the older listener group (*SD* = 12.0, range = 3.8–46.3 dB HL). Hearing sensitivity differed significantly between the younger and older adults (*t* = -7.34, *p* < 0.001). Neither in the younger group (*r* = 0.08, *p* = 0.74) nor in the older group (*r* = 0.12, *p* = 0.65) did hearing sensitivity correlate with age.

### Experimental Design

#### The VAM Corpus

The stimuli were taken from the audio-only section of the audio-visual “Vera am Mittag” (a German TV talk show; henceforth: VAM) corpus for affectively colored conversational speech (Grimm et al., 2008). The VAM corpus consists of 1018 affective utterances divided into two subsets: VAM-Audio I and VAM-Audio II. VAM-Audio I consists of 499 utterances produced by 19 different speakers (4 male and 15 female). VAM-Audio II consists of 519 utterances by 28 speakers (7 male and 21 female). The corpus comes with mean reference values for the degree of arousal and valence for each utterance. These reference values were collected with the same pictorial 5-point scales, ranging from -1 (calm/negative) to +1 (aroused/positive), as employed in the present study. Each utterance was evaluated by a group of younger adults (VAM-Audio I: 17 evaluators, VAM-Audio II: 6 evaluators). Their mean ratings are treated as reference values in our analyses. According to the reference values, the VAM corpus provides a good coverage of the emotional space (arousal: min = -0.83, max = 1.00; valence: min = -0.80, max = 0.77). However, due to the discussion topics within this TV program (relationship crises, jealousy, fatherhood questions, etc.), the emphasis within the corpus was found to be on neutral to more negative emotions (Grimm et al., 2008).

#### Subsets for the Arousal and Valence Rating Tasks

Stimuli for the affect rating experiments were selected from both VAM-Audio I and II. In order to not overload, confuse, or bias participants, the two age groups were presented with two separate one-dimensional emotion rating tasks, i.e., participants rated only one emotion dimension per stimulus at the time. Separate stimulus sets for arousal and for valence were created, whereby both sets complied with the following three criteria. First, we only selected stimuli that did not exceed the perceptual window for information integration, i.e., utterances did not exceed 3 s including hesitations and pauses (cf. Pöppel, 2004). Moreover, longer utterances might be less consistent in their degree of arousal or valence thus making it harder for the participants to attribute the utterance to one of the five steps on the scale. Second, since semantic meaning may change the emotional content of an utterance, e.g., when non-verbal information is negative while the verbal information is positive as in sarcasm (Cheang and Pell, 2008), only semantically neutral utterances were selected to minimize semantic interference (e.g., ‘*Was hast du getan*?’ ‘What have you done?,’ ‘*Erzählst denn du*’ ‘(What) do you say,’ ‘*Hab ich mir doch gedacht*’ ‘That’s what I have thought,’ ‘*Er ist relativ jung*’ ‘He is relatively young’). To that end, transcriptions of the utterances were presented to three independent evaluators who were asked whether they thought a particular utterance has a positive or negative connotation or whether it was semantically neutral. Only the utterances labeled as neutral by at least two of the three raters were included in the final stimulus sets. Third, only stimuli which had arousal or valence reference ratings closest to the values of the five steps on the scale (1, 0.5, 0, -0.5, -1) were included in the final test sets. In order to familiarize participants with the task, another four utterances per rating task were selected to serve as practice trials.

The final item set for the arousal rating task consisted of 24 utterances from 17 different speakers in total (3 male and 14 female speakers; minimum reference rating = -0.66, maximum reference rating = 0.94). The item set for the valence rating task included 18 utterances from 15 different speakers (4 male and 11 female speakers; minimum reference rating = -0.80, maximum reference rating = 0.77). Please note that as fragments were selected to represent a range of either arousal or valence reference ratings and due to the stimulus selection constraints outlined above, the stimulus sets differed for the two affect dimensions. There was, however, an overlap of two utterances between the item sets; thus, two stimuli were rated for both arousal and valence. There was a large overrepresentation of utterances with negative valence in the corpus due to the corpus’s nature, making it hard to control for valence in the arousal sentences. In fact, no stimuli for the arousal task had positive valence (valence values ranged from -0.8 to 0.1, *SD* = 0.25). The arousal values for the valence sentences were more balanced (range: -0.8 to 0.9, *SD* = 0.48).

#### Acoustic Measurements

Acoustic analyses were carried out for the stimuli. Acoustic measurements were related to the VAM reference values for the two emotion dimensions. Mean *F*_0_ and mean intensity were calculated (averaged over the phrase) using Praat (Boersma and Weenink, 2013). As a measure of tempo, articulation rate was calculated by dividing the number of syllables in the canonical transcription of the utterance by its file length excluding pauses longer than 100 ms. Spectral slope related to vocal effort is reflected in the spectral information described by the Hammarberg Index (Hammarberg et al., 1980). The Hammarberg Index is defined as the intensity difference between the maximum intensity in a lower frequency band [0–2000 Hz] versus a higher frequency band [2000–5000 Hz]. In this study, the Hammarberg Index was used as an energy distribution measure averaged across the entire utterance. **Table 1** shows the Pearson correlation coefficients for the correlations between the acoustic parameters and for the correlations of the acoustic parameters with the reference ratings for arousal and valence.

**Table 1:** Correlation coefficients per emotion dimension.

		Mean *F*_0_	Mean intensity	Articulation rate	VAM reference values
Arousal	Mean *F*_0_	-			0.47^∗∗^
	Mean intensity	0.79^∗∗∗^	-		0.75^∗∗∗^
	Articulation rate	-0.38	-0.42^∗^	-	-0.20
	Hammarberg index	0.25	0.39	-0.13	0.39^∗∗^
Valence	Mean *F*_0_	-			-0.35
	Mean intensity	0.67^∗∗^	-		0.06
	Articulation rate	-0.16	-0.21	-	0.20
	Hammarberg index	0.47^∗^	0.71^∗∗^	-0.22	0.05

*Pearson correlation coefficients between the acoustic parameters and with the reference ratings for arousal and valence for the two sets of stimuli. For VAM reference values, Kendall’s Tau was used instead of Pearson’s correlation coefficients, as reference ratings for arousal were not normally distributed in our data set. ^∗^p < 0.05, ^∗∗^p < 0.01, ^∗∗∗^p < 0.001*.

As expected, for both arousal and valence, a positive correlation between mean *F*_0_ and mean intensity was found (arousal: *r* = 0.79; valence: *r* = 0.67). Articulation rate correlated with mean intensity for the arousal stimuli (*r* = -0.42) but did not correlate with any of the other acoustic parameters, or with the reference affect ratings. For the Hammarberg index of vocal effort, we found significant, positive correlations with mean *F*_0_ (*r* = 0.47) and mean intensity (*r* = 0.71) for the valence stimuli but not for the arousal stimuli. Banse and Scherer (1996) intercorrelated many different acoustic parameters from affective speech, including mean *F*_0_, the Hammarberg Index, and a measure related to mean intensity. Our findings are in agreement with theirs in terms of the direction of the correlations, though effect sizes differ slightly: they found correlations for mean *F*_0_ with mean intensity (*r* = 0.62), Hammarberg Index with mean *F*_0_ (*r* = 0.34), and Hammarberg Index with mean intensity (*r* = 0.60).

In general, correlations between acoustic parameters and the VAM reference values were stronger for arousal than for valence (cf. also Sauter et al., 2010; Lima et al., 2013). For arousal, positive correlations were found for mean *F*_0_, mean intensity, and the Hammarberg Index with the reference ratings, as has been found in other studies (e.g., Pereira, 2000; Schröder et al., 2001; Schröder, 2006). In contrast, there were no significant correlations between the reference ratings for valence and any of the acoustic parameters. Other studies have found correlations between valence and acoustic parameters, but weaker than those for arousal (Pereira, 2000; Schröder et al., 2001; Schröder, 2006). Pereira (2000), for instance, only found a significant correlation between mean *F*_0_ and valence for male speakers (note that the majority of the speakers in our stimulus set were female).

### Procedure

Prior to testing, all participants gave written informed consent to use their data for research purposes. Younger adults were tested individually in a sound-attenuated booth at the Centre for Language Studies Lab at the Radboud University in Nijmegen. Older participants were either tested in a quiet environment at their homes or in a quiet room at a senior club house. They were comfortably seated in front of a laptop. First, participants carried out the self-paced emotion ratings tasks (15–30 min for the two rating tasks). The order in which the two emotion rating tasks (Arousal, Valence) were presented was counterbalanced across participants. Subsequently, the older participants completed the MoCA test (15 min) and hearing sensitivity of both age groups was measured (15 min). The total experiment duration was about 1 h.

Prior to each rating task, the emotion dimension at hand and the pictorial rating tool were explained to the participant. A printed version of the rating tool was provided which depicted the five steps for each emotion dimension. On the printed version, numbers from 1 to 5 were assigned to each step (arousal: very calm = 1, very aroused = 5; valence: very negative = 1, very positive = 5), replacing the values ranging from -1 to +1. The meaning of each step on the scale was described to the participant by the experimenter and the participant’s attention was particularly drawn to the changing attributes of the figure, i.e., calm versus expressive (arousal) and smiling versus frowning (valence). Each stimulus was presented twice to increase statistical power. There was no break in between the two renditions of the stimulus set. The order of the utterances was randomized for each rendition. Participants were informed that each utterance occurred twice in the experiment. Furthermore, in addition to verbal instructions, written instructions were provided on the computer screen. Throughout the instructions, participants could ask questions. Each rating task was preceded by a practice session, which was identical to the set-up of the rating task. During the practice session, four items were presented in a randomized order in two renditions. Practice items were different for the arousal and the valence task.

Each trial started with the presentation of a fixation cross (250 ms) followed by a white screen (100 ms) in order to alert participants that a stimulus was coming up. Then the utterance was presented auditorily to both ears via circumaural headphones (Sennheiser HD 215). The mean presentation level was kept constant at 70 dB SPL for both participant groups. Participants entered the number corresponding to the intended step on the scale via the keyboard and proceeded to the next trial by pressing the return key. Participants were asked to rate the utterances as intuitively as possible. They could listen to an utterance multiple times by pressing the space bar on the keyboard. This option was provided in order to allow participants to fully process the auditory input, since the utterances were relatively short. However, they were encouraged to make their ratings as spontaneously as possible, i.e., to use the repeat function only if they thought they had missed crucial information to be able to rate the utterance. Collapsed over renditions, younger adults listened to the arousal stimuli on average 1.14 times (range: 1–6) and older adults 1.18 times (range: 1–3). For the arousal rating task, 87.1% of the utterances (younger adults: 90.0%, older adults: 83.7%) were rated on their first presentation. For the valence ratings task, collapsed over renditions, younger adults listened to the stimuli on average 1.13 times (range: 1–7) and older adults 1.27 times (range: 1–3). Of the utterances, 83.6% (younger adults: 89.9%, older adults: 76.1%) were rated on their first presentation.

## Results

### Analysis

In order to investigate whether younger and older listeners use acoustic cues differently when rating affect in speech, the age groups’ ratings of affect (the dependent variable) were compared using linear mixed-effects regression analyses (with random intercepts for stimulus and participant). Note that parametric tests like regression, including linear mixed-effects models, are robust against violations of the assumption of normal distribution. Moreover, linear mixed-effect models have been shown to be good models to analyze Likert scale data (cf. Norman, 2010). Nevertheless, we also analyzed whether results obtained with our linear mixed-effect regression models were replicated in analyses for ordinal data, although there are suggestions that the risk of finding a false positive (Type 1 error) are higher for the ordinal data analysis method compared to the linear mixed-effects method (cf. Kizach, 2014). The initial model allowed for two-way interactions between each of the acoustic parameters and age group and between each of the acoustic parameters and rendition (i.e., whether they rated the stimulus for the first or the second time); the latter serving as a control variable. Moreover, an interaction effect of age group and rendition was tested. The model with the lowest Akaike Information Criterion (AIC) was considered the best-fitting model. Interactions and predictors that did not improve model fit were removed using a stepwise exclusion procedure (interactions before simple effects, and those with the highest non-significant *p*-values first).

The second research question, concerning the impact of hearing sensitivity on the affect ratings, was investigated using the data from the group of older adults only, where differences in individual hearing sensitivity were more pronounced (see Section “Participants”). Therefore, in a second analysis, hearing sensitivity was associated with the affect ratings by the older adults group. The set-up and model selection procedure of this analysis was similar to the first analysis except for the continuous hearing sensitivity measure (PTA) replacing the binomial age group factor in the previous analysis. Analyses were carried out for the arousal and the valence tasks separately. **Table 2** lists the mean arousal and valence ratings, with standard deviations, for the younger and older adults separately.

**Table 2:** Mean arousal and valence ratings, with standard deviations, for the younger and older adults separately.

	Younger adults		Older adults	
	Mean	*SD*	Mean	*SD*
Arousal	-0.044	0.65	0.062	0.60
Valence	-0.013	0.60	0.037	0.60

### Analysis of Arousal Rating

**FIGURE 1:**
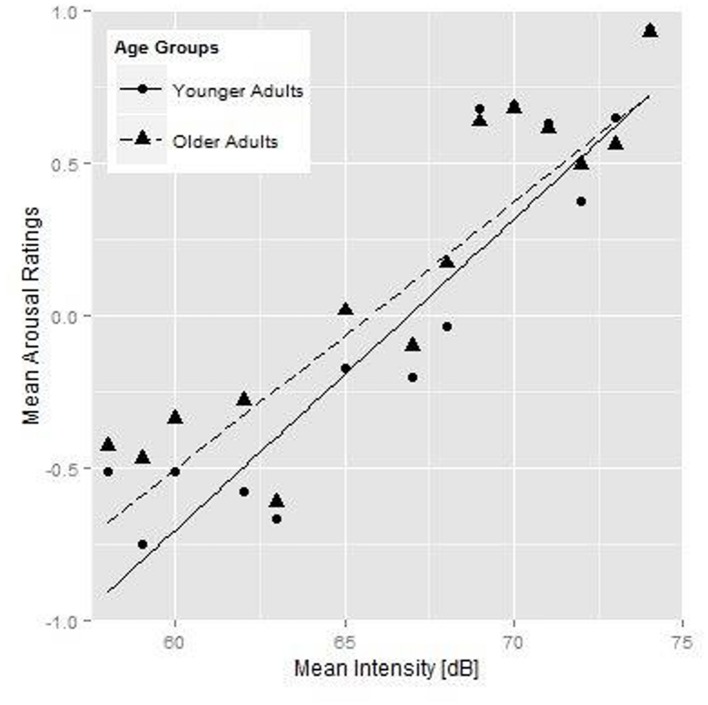
**Younger (round symbols) and older participants’ (triangular symbols) mean arousal ratings for each individual stimulus as a function of mean intensity of the speech fragments, and the fit lines for the younger (solid line) and older (dashed line) participants**.

**Figure 1** shows the relationship between mean intensity and the arousal ratings; more particularly the mean arousal ratings per stimulus for the younger (round symbols) and older (triangles) listener groups plotted against the mean intensity (on the *x*-axis). **Tables 3** and **4** show the best-fitting models for the two arousal analyses. Both younger and older adults associated higher mean intensity with a higher level of arousal (see the significant simple effect for mean intensity in **Table 3**), which is also shown by the upward sloping fit lines in **Figure 1** (solid lines for the younger and dashed lines for the older participants). There was no general effect of age group in the arousal rating task (cf. **Figure 1**). If we were to remove the acoustic variables from our arousal rating analysis to only test for a simple age group difference across renditions, the Age Group effect also fails to reach significance (β = 0.106, *SE* = 0.058, *p* = 0.077). However, the older adults showed a less steep intensity increment (as shown by the interaction between Age Group and mean intensity), i.e., the older adults showed a smaller effect of mean intensity on their ratings than the younger adults. Moreover, we found a significant interaction between Age Group and Rendition, i.e., older participants rated the second rendition of the stimulus as more aroused. Importantly, acoustic measures for mean *F*_0_, articulation rate, and the spectral measure were not predictive of participants’ ratings in the arousal task.

**Table 3:** Fixed effect estimates of the best-fitting models of performance for the group comparison of the arousal data; bold indicates significant results, number of observations = 1776, AIC = 1496.

	β	*SE*	*p*
Age group	0.065	0.061	0.29
Rendition	0.024	0.022	0.28
**Mean intensity**	**0.102**	**0.011**	**<0.001**
**Age Group × rendition**	**0.083**	**0.033**	**0.012**
**Age Group × mean intensity**	-**0.014**	**0.004**	**<0.001**

**Table 4:** Fixed effect estimates for the best-fitting models of performance for the analysis of the arousal data for the older adults only; bold indicates significant results, number of observations = 816, AIC = 804.6.

	β	*SE*	*p*
**Rendition**	**0.107**	**0.026**	**<0.001**
**Mean intensity**	**0.087**	**0.011**	**<0.001**

The analysis of the older adults’ data (**Table 4**) to investigate the role of hearing sensitivity showed a similar picture to the age group comparison: Only significant effects for mean intensity and rendition were found. Thus, again, stimuli were rated as more aroused when rated for the second time and higher mean intensity was perceived as more aroused among the older group. Importantly, however, there was no simple effect of hearing sensitivity, nor was there an interaction between hearing sensitivity and interpretation of the acoustic measures^1^.

### Analysis of Valence Ratings

**FIGURE 2:**
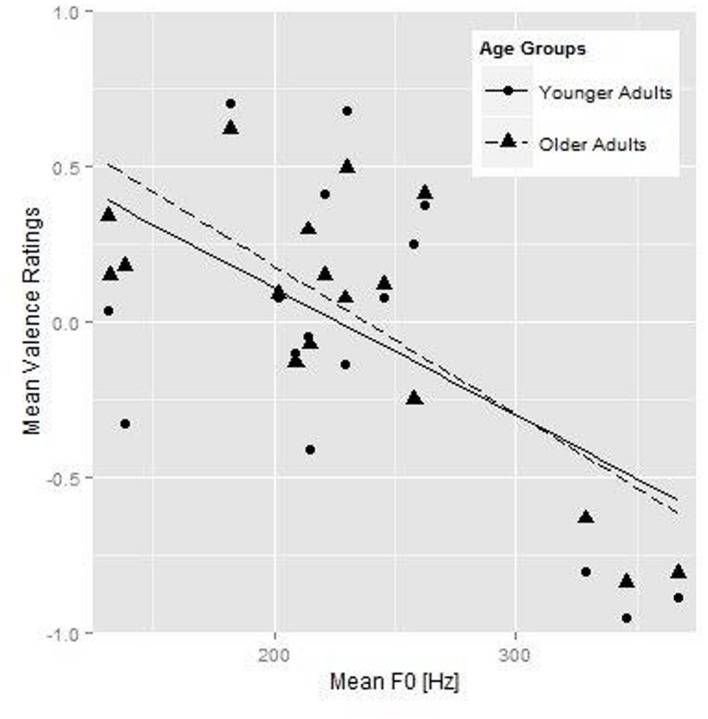
**Younger (round symbols) and older participants’ (triangular symbols) mean valence ratings for each individual stimulus as a function of mean *F*_0_ of the speech fragments, and the fit lines for the younger (solid line) and older (dashed line) participants**.

**Figure 2** shows the relationship between mean *F*_0_ and the valence ratings in terms of the mean valence ratings per stimulus for the younger (round symbols) and older (triangles) listener groups plotted against the mean *F*_0_ (on the *x*-axis). Fit lines for the younger (solid line) and older (dashed line) participants are also shown. **Tables 5** and **6** show the best-fitting models for the two valence analyses. The age group comparison for valence (**Table 5**) showed a simple effect for mean *F*_0_. Higher mean *F*_0_ of the stimuli was associated with more negative utterances in younger adults. The significant interaction between Age Group and mean *F*_0_ indicates that both age groups rated higher mean *F*_0_ as more negative, but the change in valence rating associated with each unit increase in F0 was larger for the older than for the younger adults (as also shown by the steeper slope of the fit line for the older adults in **Figure 2**). Rendition, mean intensity, articulation rate, and the spectral measure of vocal effort were not predictive of valence ratings, nor did they interact with age group.

**Table 5:** Fixed effect estimates of the best-fitting models of performance for the group comparison of the valence data; bold indicates significant results, number of observations = 1332, AIC = 1415.

	β	*SE*	*p*
**Mean *F*_0_**	-**0.004**	**0.001**	**0.008**
Age Group	0.050	0.042	0.24
**Age Group × mean *F*_0_**	-**0.001**	**3.271 × 10**^-^**^4^**	**0.038**

**Table 6:** Fixed effect estimates for the best-fitting models of performance for the analysis of the valence data for the older adults only; bold indicates significant results, number of observations = 612, AIC = 767.8.

	β	*SE*	*p*
**Mean *F*_0_**	-**0.005**	**0.001**	**<0.001**
**Hearing sensitivity**	**0.006**	**0.002**	**0.018**
**Hearing sensitivity × mean *F*_0_**	-**5.795 × 10^-05^**	**2.117 × 10^-05^**	**0.006**

The analysis of the older listeners’ valence data (**Table 6**) to investigate the role of hearing sensitivity showed a simple effect for mean *F*_0_. As was found in the age group comparison, higher mean *F*_0_ lead to more negative ratings. Importantly, there was a significant simple effect of hearing sensitivity: poorer hearing (i.e., higher PTA values) was associated with more positive valence ratings. Finally, there was an interaction between hearing sensitivity and mean *F*_0_: the change in valence rating associated with each unit increase in F0 was larger with increasing hearing sensitivity^2^.

## Discussion

Previous research revealed age differences in the perception of verbal affect (e.g., Orbelo et al., 2005; Paulmann et al., 2008). The current study investigated the origin of this age difference. The first aim was to investigate whether younger and older listeners differ in the way they make use of affect-related acoustic cues in natural speech; more specifically, mean *F*_0_, mean intensity, articulation rate, and vocal effort. The second aim was to determine the impact of age-related hearing sensitivity differences on the use of these affective cues. Three methodological aspects were combined to investigate the perception of affect: the perception of acoustic parameters was linked to individual hearing sensitivity, conversational (rather than acted) speech was used in the rating tasks, and participants rated two emotional dimensions, arousal and valence, rather than classified affect categories.

The results showed that only two acoustic cues predicted our participants’ ratings. Both the younger and the older age groups associated higher mean intensity with an increased level of arousal and associated higher mean *F*_0_ with more negative valence. As pitch is considered the most telling component of affective prosody (Mozziconacci, 1998; Hammerschmidt and Jürgens, 2007; Rodero, 2011), the finding that mean *F*_0_ was a good predictor of valence was not surprising. Others have found, however, that higher levels of arousal are also particularly related to higher pitch (Schröder, 2006). This was not borne out by our data and may be accounted for by the interplay between several acoustic cues: Mean *F*_0_ and mean intensity were highly correlated in the selected subset of arousal stimuli. For the present item sample, mean intensity was probably the most prominent acoustic cue and was therefore a better predictor for arousal ratings than mean *F*_0_. It is unclear why intensity differences were a stronger cue to arousal than vocal effort, as the former, but not the latter, could simply relate to the speaker’s distance from the microphone. Possibly, listeners rely on these salient and prototypical intensity differences because vocal effort may be more difficult to compare across multiple speakers in a situation in which a listener has to evaluate affect across multiple speakers (as is the case in our design).

The arousal data showed an effect of rendition in that the older (but not the younger) adults rated the same utterances as more aroused in the second rendition. Note that this effect was absent in the valence ratings. This finding suggests that rating behavior can change over the course of a rating task, in older participants in particular, which should encourage researchers to investigate block or rendition effects in their experimental designs. Possibly, listening to affective utterances raises the general level of arousal within the listener, thus slowly increasing the reference or resting level for arousal over time.

While age-related differences in the perception of emotion categories in speech are well documented (e.g., Orbelo et al., 2005; Paulmann et al., 2008; Mitchell et al., 2011), no such difference was observed with the dimensional approach in the current study. In other words, in our study, younger and older adults did not generally differ in their ratings of the emotion dimensions arousal or valence. This absence of a general age difference in our study could be due to lack of statistical power (given our relatively small sample size), or to our use of the dimensional approach instead of classification of emotion categories, even though age differences have been reported using that approach as well (e.g., Lima et al., 2014). Nevertheless, we found age differences in the use or interpretation of both mean intensity and mean *F*_0_, which was the focus of our study. For arousal, older adults’ ratings were less affected by changes in mean intensity compared to younger adults. We will come back to this point below. For valence, differences in mean *F*_0_ affected the ratings of older adults *more* than those of younger adults. Hence, the effect of a mean *F*_0_ change on valence rating was more pronounced for the older than for the younger adults. Our finding may relate to a recent study by Lima et al. (2014). Lima et al. (2014) investigated affect perception in younger and older adults using ‘affective bursts,’ which are vocalizations without verbal content, such as laughter, sobs, and sighs. Their study showed that pitch was used differently across age groups depending on whether age groups evaluated positive or negative affect. In Lima et al. (2014), mean *F*_0_ was a stronger predictor for rating fear and sadness (negative valence) in older adults than in younger adults. Conversely, F0 differences were associated with pleasure (positive valence) in younger but not in older adults. As our set of valence stimuli was skewed toward negative affect, our finding that older adults were more sensitive to F0 differences in the interpretation of valence agrees with their findings for fear and sadness. Thus, both younger and older adults used mean *F*_0_ as a cue to valence, but the age groups used mean *F*_0_ to a different extent. Nevertheless, as observed before, this differential use of mean *F*_0_ as a cue to valence did not lead to age differences in overall valence ratings. As argued earlier and below, the correlations between valence ratings and the acoustic cues were low. Possibly, older adults and younger adults also differed in their use of other, here not investigated, acoustic cues that cue valence, which counteract the differences in the use of mean *F*_0_.

Previous research has shown that hearing loss impacts intensity discrimination (Boettcher et al., 2001). Considering that mean intensity was identified as the main cue for rating arousal in the current study, deterioration in the perception of intensity due to age-related hearing loss may account for the observed age differences in the arousal rating task. However, this was not confirmed by the analysis of the older adults’ data: Among older adults, individual hearing loss was not related to ratings of arousal. This confirms recent findings by Dupuis and Pichora-Fuller (2015) who also found that emotion categorization accuracy by younger and older adults was not correlated with their auditory abilities (i.e., neither with their hearing sensitivity, nor with measures of auditory processing, such as F0 or intensity difference limens). There are several possible interpretations of this finding. Older adults may be less willing than younger adults to use the entire rating scale while performing a rating task. As also argued by Lima et al. (2014), this ‘conservatism’ account is somewhat unlikely, however, considering that the older adults used a wider range of the scale than younger adults for the valence task. A second explanation could be that arousal perception is relatively robust against mild-to-moderate hearing loss because arousal is cued by several other acoustic parameters. In both the current study and previous work (Pereira, 2000; Schröder, 2006), arousal has been reported to show strong correlations with multiple acoustic parameters, including intensity and pitch measures. Hence, possibly, the perception of arousal in affective speech is more robust against mild sensory degradations due to the availability of clear acoustic cues which reliably signal arousal in the speech signal. Hearing loss might have played a role if our older adult sample had been (even) more diverse in hearing sensitivity. Third, the interaction between the use of mean intensity and age group may still have an auditory/perceptual origin: possibly, age-related hearing decrements in auditory processing that are not apparent from the tone audiogram may relate to older adults’ smaller-sized intensity effect. Finally, age differences in affect perception may be dissociated from hearing loss if they primarily arise at processing levels following auditory analysis. This account would be in keeping with the meta-analysis by Ruffman et al. (2008) that age differences in emotion recognition arise due to age-related changes in the “social brain,” i.e., due to changes in volume of frontal and temporal brain areas, as well as changes in neurotransmitters. In other words, even if older adults are able to hear cues that signal affect, higher-order processing of these cues may result in less differentiation of emotional content than in younger adults.

Individual hearing sensitivity did, however, impact the interpretation of valence in our experiment, i.e., poorer hearing generally led to more positive valence ratings. This observation makes it less likely that the lack of a hearing sensitivity effect on arousal (discussed above) should be attributed to lack of statistical power due to a relatively small sample of older adults. In contrast to our findings, Orbelo et al. (2005) did not find such a global effect of hearing loss on the comprehension of affective prosody, even though the mean and standard deviation of their subjects’ hearing sensitivity was comparable to the hearing loss observed in the present study. The difference in findings may, however, originate from a difference in the used materials. Orbelo et al. (2005) used acted affective speech material, whereas we used natural affective speech. As argued in the introduction, the more prototypical acoustic expression in acted compared to natural speech may lead to a more extreme realization of affective prosody (Scherer, 1986; Wilting et al., 2006), which may be relatively easy to perceive, even for people with hearing loss (Grant, 1987). With natural, and hence less extreme speech materials, as used in our study, those with poorer hearing may be less certain about their valence perception.

There was no impact of hearing sensitivity on mean intensity, articulation rate, or the spectral measure of vocal effort on the participant’s valence ratings. This was not unexpected as none of these parameters predicted valence in general. Importantly, apart from the general hearing loss effect of valence rating, hearing loss also modulated listeners’ use of the pitch cue for valence. Based on findings of poorer pitch discrimination in older compared to younger adults (He et al., 2007; Souza et al., 2011), one would expect pitch to affect ratings of older adults less than those of younger adults. We can only speculate on why our findings show the opposite result. Note again that mean *F*_0_ showed up as a significant predictor of valence ratings in our study, but **Table 1** showed no correlation between mean *F*_0_ and the reference ratings that came with the conversational speech corpus. This shows that the relationship between the valence ratings and the acoustic measures we focused on here was not as strong and straightforward as for arousal. Participants in the current study relied on mean *F*_0_ when rating valence. However, the acoustic profile of affective speech is complex and is not only encoded in pitch, intensity, and tempo of the utterance. Some variations in affective speech may be captured by alternative, perhaps more subtle, cues (Bänziger et al., 2014) that we did not include here. Voice quality, for example, is known to be used in verbal affect perception (Grichkovtsova et al., 2012), and is related to the perception of valence in affective speech (Waaramaa and Leisiö, 2013). Possibly, alternative cues for valence, such as voice quality, may have been less available to the older listeners with poorer hearing in our experiment, leading to a differential use of mean *F*_0_ in the current sample of older adults. Note also that this may then tie in with the account provided above on the similarity between our valence results and those by Lima et al. (2014). A different weighing of acoustic cues across age groups may result from age-related hearing loss, but not necessarily. Lima et al. (2014) found that age groups were equally efficient in using acoustic cues but that there were differences in the patterns of emotion-specific predictors. Lima et al. (2014) therefore argue, in line with Ruffman et al. (2008), that age-related differences in weighting of acoustic cues may reflect changes in higher-order processing. Clearly, follow-up research with more controlled or experimentally manipulated materials would be required to test this cue trading in more detail, and to see to what extent changes in cue use are driven by age-related changes in perception or in higher-order processing.

This study showed that both younger and older listeners base their affect ratings on acoustic cues in speech: mean intensity for arousal and mean *F*_0_ for valence. However, the extent to which these acoustic parameters are used for affect rating varies across age groups: intensity differences are used less by older adults for arousal ratings, while differences in mean *F*_0_ influence valence ratings by older adults more than they do those of younger adults. Arousal perception seems to be robust against mild-to-moderate hearing loss which may be explained by the availability of multiple clear acoustic parameters consistently signaling arousal.

## Conclusion

This study suggests that age differences in the perception of affect relate to differences in acoustic cue use, and that age differences in cue use can only partly be explained by age-related changes in hearing sensitivity. Moreover, differences in cue use for the two emotion dimensions suggest that future studies should treat the perception of arousal and valence separately.

## Author Contributions

JS, EJ, and OS were substantially involved in the design, the data collection, the analysis and interpretation, and the drafting of the current work. JS took leading role in the design, the data collection, analysis and drafting the work. EJ and OS critically revised the work by JS, contributed to the interpretation and supported the writing process. Finally, all authors approved the version for publication.

## Conflict of Interest Statement

The authors declare that the research was conducted in the absence of any commercial or financial relationships that could be construed as a potential conflict of interest.

## Footnotes

1 Analyses of the group comparison and the data of the older adults with a statistical method specifically for ordinal data (cumulative link mixed models, CLMMs, cf. Agresti, 2002) showed similar results to those obtained with the linear mixed-effect models.

2 Analyses of the group comparison and the data of the older adults with CLMMs showed similar results to those obtained with the linear mixed-effect models, except for the interaction between Age Group and mean *F*_0_, which became marginal (β = -0.003, *SE* = 0.002, *p* = 0.06).
